# The longer, the better? Optimizing dystrophin constructs for cardiac gene therapy in Duchenne muscular dystrophy

**DOI:** 10.1016/j.omta.2026.201870

**Published:** 2026-11-02

**Authors:** Agnieszka Łoboda, Józef Dulak

**Affiliations:** 1 Department of Medical Biotechnology, Faculty of Biochemistry, Biophysics and Biotechnology, Jagiellonian University, Gronostajowa 7, 30-387 Kraków, Poland

## Main text

Gene therapy for Duchenne muscular dystrophy (DMD) has so far focused mainly on improving skeletal muscle function; however, effective treatment of the disease will also require protection of the heart. In a recently published study in *Molecular Therapy Advances*, Keegan et al.^1^ addressed this challenge using a patient-in-a-dish model based on induced pluripotent stem cell-derived cardiomyocytes (iPSC-CMs), as discussed in the following text.

DMD is an X-linked genetic disease caused by mutations in the *DMD* gene, with more than 7,000 pathogenic variants reported, affecting about 1:5,000 newborn boys.^2^ The absence of dystrophin, the muscle structural protein encoded by the largest known human gene, impairs the function of skeletal, respiratory, and heart muscles. The disease phenotype, characterized by progressive difficulties with walking, usually becomes evident between 2 and 3 years of age. Patients lose ambulation during adolescence, and progression of cardiomyopathy leads to heart failure, which is the major cause of premature death; however, thanks to contemporary multidisciplinary care, an increasing proportion of patients survive into their 30s and 40s.

The disease remains incurable. Gene replacement strategies are challenging due to the large size of the *DMD* gene (for reviews, see Duan et al.^2^ and Loboda et al.^3^). The 12-kb dystrophin coding sequence exceeds the approximately 4.7-kb packaging capacity of adeno-associated viral (AAV) vectors, which are currently considered the most suitable for muscle and cardiac gene delivery. Consequently, gene therapy approaches use shorter dystrophin constructs known as microdystrophins. This concept comes from observations of some Becker muscular dystrophy (BMD) patients who, despite deletion of a significant part of the *DMD* gene, express truncated dystrophin that can preserve ambulation into late adulthood.^3^ Nevertheless, even these BMD dystrophins are still too large for the AAV. Shorter dystrophins can also be restored by four mutation-specific antisense oligonucleotides (ASOs), which have received accelerated approval in the USA but not in Europe, although their overall clinical benefit remains unverified.^3^ Moreover, the FDA has approved delandistrogene moxeparvovec-rokl (Elevidys), an AAV-based microdystrophin gene therapy, for ambulatory patients aged 4 years and older with a confirmed *DMD* mutation. However, its use is currently accompanied by a boxed warning regarding serious liver injury and acute liver failure, including fatal cases. Beyond ongoing debate about its effects on muscle function,^4^ the crucial issue remains targeting the heart. This aspect is difficult to address in commonly used DMD animal models, which do not fully recapitulate human cardiac disease.^2^^,^^3^ Therefore, recently, iPSC-CMs have increasingly been used to investigate DMD cardiomyopathy and test potential therapeutic approaches.

To assess the cardioprotective effects of dystrophin constructs, Keegan et al.^1^ applied iPSC-CMs derived from three distinct genetic backgrounds (Figure 1). One DMD line was generated by CRISPR-mediated engineering of healthy cells to introduce a mutation resulting in loss of the N-terminal dystrophin region encoded by exons 1–6, recapitulating dystrophin truncation associated with early-onset dilated cardiomyopathy. The other two lines were derived from DMD patients, with corresponding isogenic control lines generated by CRISPR-mediated correction of the respective *DMD* mutations. Using these models, the authors compared three different microdystrophin variants, microDYS1 (147 kDa), microDYS2 (135 kDa), and microDYS3 (138 kDa), representing constructs developed for DMD gene therapy by Solid Biosciences, Pfizer, and Sarepta Therapeutics, respectively. They also evaluated a substantially larger Becker-derived minidystrophin construct (miniDYS, 222 kDa) containing additional functional domains. To minimize variability in AAV-mediated transgene expression, the authors modified the iPSC-CMs (at day 10 of differentiation) with lentiviral vectors, allowing permanent expression through transgene integration. A zeocin-resistance cassette enabled selection of transduced CMs, as both the dystrophin transgene and selection marker were driven by the CM-specific troponin T promoter. By establishing stable transduction, the authors sought to achieve comparable transgene expression levels; however, microDYS3 (the Sarepta construct) showed lower expression than the other dystrophins.

**Figure 1:**
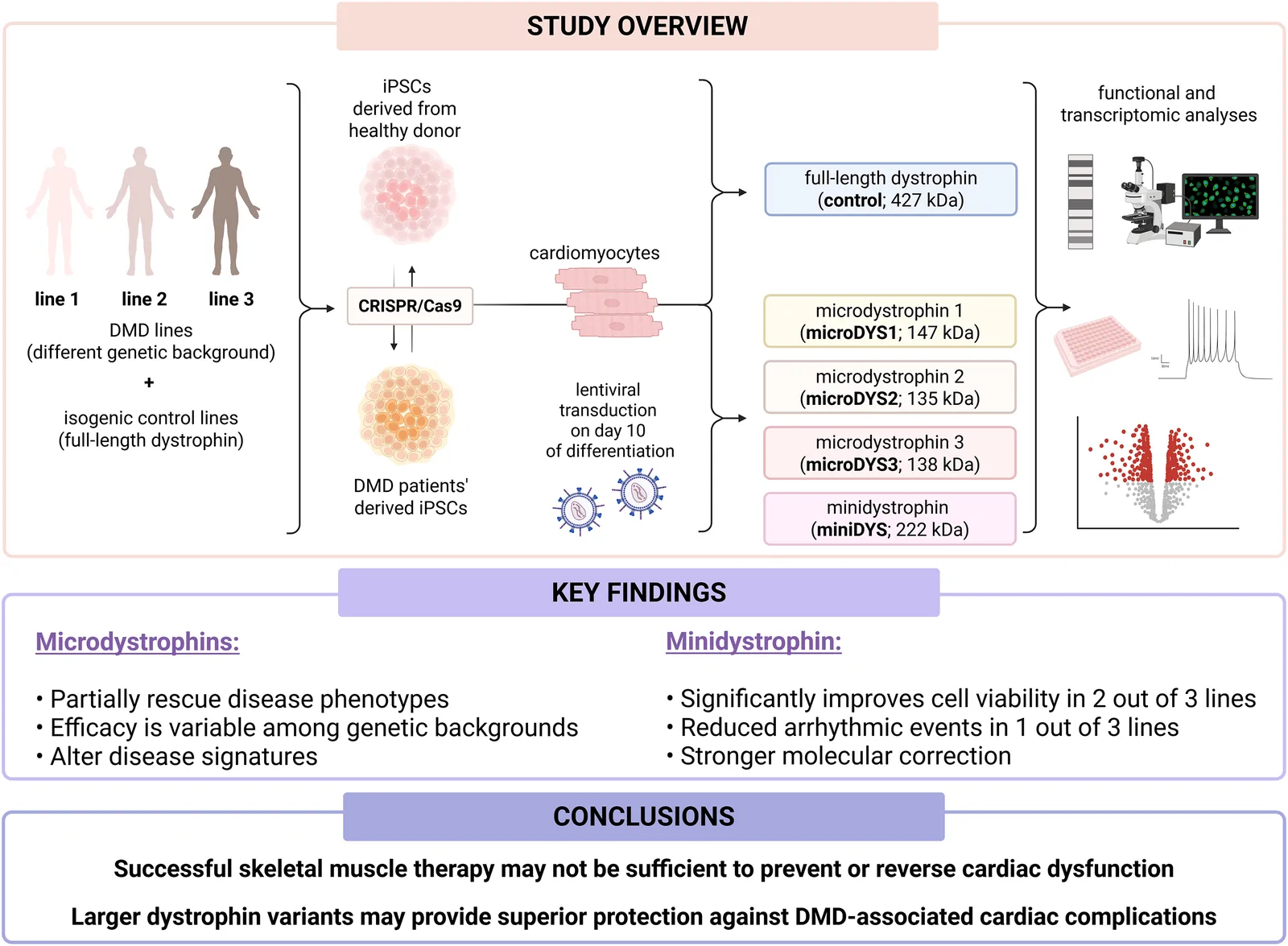
Schematic overview of the experimental approach, key findings, and therapeutic implications of the work by Keegan et al.^1^

Dystrophin restoration partially rescued the reduced cell and nuclear size observed in DMD cardiomyocytes, although the effect varied across genetic backgrounds and dystrophin constructs. Bulk RNA sequencing (RNA-seq) revealed alterations in the gene expression profile after microdystrophin or minidystrophin expression; however, the expression profiles did not reach the level observed in control, healthy iPSC-CMs. While dystrophin deficiency results in arrhythmic activity and abnormalities in calcium handling, microdystrophins did not normalize arrhythmia, whereas minidystrophin significantly reduced the frequency of arrhythmic events in one genetic background. However, some calcium-handling parameters were improved by both microDYS3 and minidystrophin, with effects varying between genetic backgrounds and endpoints. These observations were consistent with analysis of calcium metabolism gene expression assessed by RNA-seq. Finally, the authors determined whether restoring dystrophin expression improved CM viability. Again, the significant effect was observed only in cells overexpressing minidystrophin, but not microdystrophins, and only in two of the three cell lines (Figure 1).

Importantly, several experimental considerations should be taken into account when interpreting these findings and assessing their relevance to therapeutic applications. First, the study highlights both the utility and the challenges of iPSC-CM models, as data obtained from a limited number of iPSC lines can be burdened by line-specific characteristics. In the work by Keegan et al.,^1^ one model expresses an N-terminally truncated dystrophin protein, which may influence cellular phenotypes, while another line did not display a significant baseline arrhythmia phenotype. It therefore remains to be determined whether such cellular variability could be addressed by investigating a larger number of independent iPSC lines or/and by using more complex models, such as 3D cardiac spheroids/organoids. Finally, the immature nature of iPSC-CMs raises additional concerns regarding their ability to fully recapitulate the phenotype of mature human cardiomyocytes. Second, the authors used lentiviral vectors, thereby avoiding the cell-to-cell variability in AAV transduction that can significantly affect therapeutic effectiveness. Third, dystrophin was delivered at an early stage of cell differentiation. Consequently, the effects of such early intervention may differ substantially from those observed in a few-year-old patients receiving systemic AAV-mediated gene therapy. Limitations also include variation in microdystrophin and minidystrophin expression levels, which make it difficult to distinguish the relative contributions of construct design, dystrophin length, and expression level to the observed functional differences.

Various microdystrophin constructs can have distinct effects in skeletal muscle and cardiac tissue. Notably, the study highlights the importance of transgene size and the presence of specific domains, as the larger minidystrophin generally outperformed the microdystrophin constructs. However, the lack of a dose-response analysis is an important limitation, particularly given previous reports of potential cardiotoxicity from high levels of microdystrophin expression and from selected microdystrophin variants.^5^^,^^6^^,^^7^ Ultimately, these findings emphasize that a successful DMD gene therapy cannot be optimized solely for skeletal-muscle transduction and functional improvement, but it must also deliver a sufficiently functional dystrophin to the heart at an appropriate, rather than supraphysiological, level.

Overall, despite these shortcomings, this study supports the potential value of dystrophin constructs that retain a greater number of functional domains and more closely resemble the native protein for the restoration of skeletal and cardiac muscle function. Importantly, recent preclinical studies using split-intein AAV-based gene therapy approaches have demonstrated the feasibility of delivering substantially larger dystrophin constructs, including full-length dystrophin, functionally superior to microdystrophins.^8^^,^^9^ However, the potential toxicity associated with high vector doses remains a concern in clinical applications. Another approach could deliver full-length mRNA using lipid nanoparticles or extracellular vesicles,^10^ but it would also need to address the challenge of repeated injections and targeting both skeletal muscle and the heart.

In summary, Keegan et al.^1^ add new questions to the ongoing debate about the effectiveness of DMD gene therapy, particularly its ability to protect the heart. In this regard, more research in better-suited models, including experimental animals and 3D human iPSC-derived models, is necessary.

## Acknowledgments

The authors are supported by the National Science Centre grants MAESTRO [2018/30/A/NZ3/00412] and DAINA [2024/52/L/NZ3/00142] (J.D.) and OPUS [2019/35/B/NZ3/02817] (A.Ł.). Figure 1 was created with BioRender.com.

## Declaration of interests

The authors declare no competing interests.
